# The Development of an Open Hardware and Software System Onboard Unmanned Aerial Vehicles to Monitor Concentrated Solar Power Plants

**DOI:** 10.3390/s17061329

**Published:** 2017-06-08

**Authors:** Francisco Javier Mesas-Carrascosa, Daniel Verdú Santano, Fernando Pérez Porras, José Emilio Meroño-Larriva, Alfonso García-Ferrer

**Affiliations:** Department of Graphic Engineering and Geomatics, Campus de Rabanales, University of Córdoba, Córdoba 14071, Spain; p12vesad@uco.es (D.V.S.); perezporras.fernando@gmail.com (F.P.P.); ir1melaj@uco.es (J.E.M.-L.); agferrer@uco.es (A.G.-F.)

**Keywords:** UAV, monitoring, open hardware, concentrated solar power

## Abstract

Concentrated solar power (CSP) plants are increasingly gaining interest as a source of renewable energy. These plants face several technical problems and the inspection of components such as absorber tubes in parabolic trough concentrators (PTC), which are widely deployed, is necessary to guarantee plant efficiency. This article presents a system for real-time industrial inspection of CSP plants using low-cost, open-source components in conjunction with a thermographic sensor and an unmanned aerial vehicle (UAV). The system, available in open-source hardware and software, is designed to be employed independently of the type of device used for inspection (laptop, smartphone, tablet or smartglasses) and its operating system. Several UAV flight missions were programmed as follows: flight altitudes at 20, 40, 60, 80, 100 and 120 m above ground level; and three cruising speeds: 5, 7 and 10 m/s. These settings were chosen and analyzed in order to optimize inspection time. The results indicate that it is possible to perform inspections by an UAV in real time at CSP plants as a means of detecting anomalous absorber tubes and improving the effectiveness of methodologies currently being utilized. Moreover, aside from thermographic sensors, this contribution can be applied to other sensors and can be used in a broad range of applications where real-time georeferenced data visualization is necessary.

## 1. Introduction

**Figure 1:**
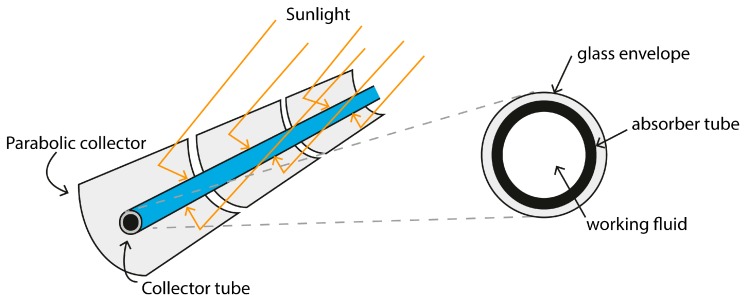
Illustration of functioning parabolic collector and absorber tube.

The constantly growing global energy demand and increasing effects of climate change have resulted in the promotion of renewable energy sources to reduce CO_2_ levels [1]. Solar technologies are a promising renewable resource because of their ever-increasing output efficiencies and ability to be used in a variety of locations [2]. Solar energy can be converted into electrical energy with different technologies such as photovoltaic (PV) panels [3], concentrated solar power (CSP) [4] and concentrator photovoltaics (CPV) [5]. Among them, CSP plants are a promising source of renewable energy to be used for predictable utility-scale power generation [6]. CSP plants utilize heat generated by solar irradiation concentrated on a small area. Currently, there are four available CSP technologies: parabolic trough collectors (PTC), solar power towers (SPT), linear Fresnel reflectors (LFR) and parabolic dish systems (PDS). Of the four, PTC is the most commercialized CSP technology to date [7], focusing direct solar radiation onto a focal line of the collector’s axis [8]. In a PTC CSP plant, the mirrors concentrate the sun’s rays on absorber tubes and the working fluid, flowing inside the tubes, absorbs solar energy by convection heat transfer. To reduce heat losses, the absorber tubes are enclosed by an anti-reflective coated borosilicate glass envelope [9] (Figure 1). Owing to weather conditions and extremely non-uniform heat flux, absorber tubes are subjected to thermal stress resulting in the rupturing of the glass envelopes, causing heat loss [10]; this presents a problematic challenge. Papaelias et al. (2016) [6] analyzed and evaluated non-destructive inspection techniques for CSP plants. These techniques are grouped into liquid penetrant inspection (LPI), magnetic particle inspection (MPI), magnetic flux leakage (MFL) and visual inspection (VI). LPI is a visual technique based on spreading special dyes over the area for inspection. It is very sensitive to small defects and the inspection is fast, but due to the large quantity of components in a plant, a considerable amount of time is needed for thorough inspection. The MPI technique magnetizes the surface area of the tubes, which has been previously sprayed with ferrous particles for inspection. The MFL technique is based on magnetizing the ferrous components for inspection with a magnetic field. Finally, VI of structural components is performed by personnel walking through the plant or by pipe-crawling inspection robots. LPI and MPI techniques cannot be used for absorber tube inspection due to the presence of the glass envelope and MFL is not suitable for inspecting absorber tubes of austenitic stainless steel alloy. VI is also ineffective as there are several kilometers of absorber tubes in a plant to be inspected. However, as the thermal losses are correlated directly with the glass of the absorber tubes, which can be measured using a thermal sensor [11,12], a more efficient alternative to VI inspection is to use robots. In CSP plants, depending on the inspection requirements, these robots can be either ground or climbing robots [13]. VI inspections use mainly RGB sensors to evaluate geometrical characteristics [14,15] and thermal sensors to detect heat loss [16,17].

In the last decade, professionals and researchers’ interest in using unmanned aerial vehicles (UAVs) to replace human activities of a dirty, dangerous and/or dull nature has increased [18]. In this context, the use of UAVs has become widespread in civil applications such as precision agriculture [19,20], emergencies [21], archaeology [22,23], traffic monitoring, [24,25] and visual inspection, [26,27] among others. Relative to industrial inspection, UAVs have been successfully used in monitoring power lines [28], gas pipelines [29] and photovoltaic plants [30]. To our knowledge, no detailed investigation has been conducted regarding the use of UAVs to monitor absorber tube heat loss in CSP plants using PTC technology.

To date, the main problems of current implementations of UAVs are the limited flight autonomy and the size-to-payload ratio [31]. Recent progress in electronics, wireless communications and the production of small-sized sensors offer new opportunities to monitor and control crops, cities, homes and the environment [32]. In this context, the design and implementation of a light-weight payload for UAVs can facilitate CSP plant inspections [33,34]. While there are complete thermographic systems already integrated with UAVs, they are not only expensive, but also closed systems that have little capacity for modification and variation [35]. Open source hardware (OSH) can be an alternative to closed systems when customization is necessary. Although OSH is quite new compared to open source software (OSS) [36], it is already being used for different applications in agriculture [37], management sensors [38], monitoring heritage buildings [39] and chemical instrumentation [40], among others. In reference to UAVs, some researchers have developed unmanned aerial platforms based on OSH autopilots [41,42] and others have used OSH to integrate and use sensors for data-collection needs [43].

This manuscript describes a system that integrates information registered by a thermographic sensor and geolocation information from OSH components to be accessible in real time by users independently of the type of device used to monitor the inspection (laptop, smartglasses, smartphone or tablet) and its operating system. Its practicality is demonstrated in its application of monitoring absorber tube heat loss in a PTC CSP plant.

The article is divided in the following sections: in Section 2, we present the system; in Section 3, we describe the technical implementation; in Section 4, some field tests and their results are presented, followed by a conclusion.

## 2. System Overview

**Figure 2:**
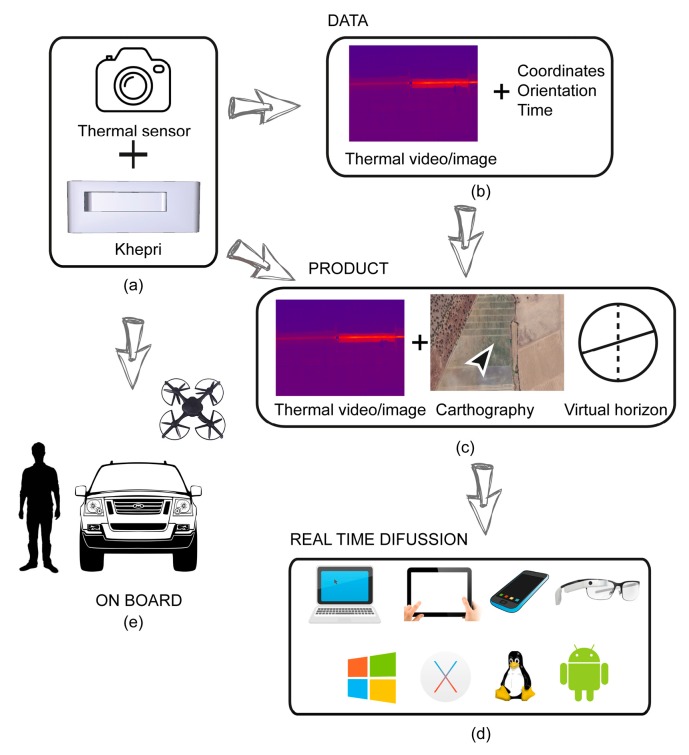
Conceptual model of the system: (**a**) Thermal sensor connected to the Khepri; (**b**) Data package generated by the system; (**c**) Thermal information and georeferentiation of the system over cartograph; (**d**) Real-time diffusion regardless of device and operating system and (**e**) Working onboard multiple platforms.

Figure 2 shows the conceptual model of the system. It has two main components (Figure 2a): the main system, and a camera sensor connected to the developed device, called Khepri, using OSH. In this project, a thermal sensor was used, but it is possible to use Red-Green-Blue (RGB) or multispectral sensors for other applications. Meter accuracy is sufficient enough to geolocate the thermographic video, as it allows the thermal sensor to be placed over the Khepri. Both components work synchronously to generate two principal data packages (Figure 2b). The first package is the thermal video registered by the sensor, and the second is the georeferenced information generated by the Khepri. These two data packages are processed to display three windows on a device screen (Figure 2c). The first window shows the information registered by the thermal sensor, the second georeferences the system on a cartograph, and the third shows an artificial horizon using orientation information. With this information, users will be able to locate incidents at the plant once they are detected through the position and orientation provided by the Khepri.

The information presented in the windows (Figure 2c) is generated and diffused in real time and accessible by various devices (laptop, tablet, smartphone, smartglasses) regardless of the operating system (Figure 2d). Finally, the main system can be used as the payload of an UAV, as well as installed on a terrestrial vehicle or used by personnel (Figure 2e). The electronic components of the Khepri were chosen after considering the restrictive load capacity of the three aforementioned platforms in both weight and dimensions. All selected components were based on OSH.

### 2.1. Selection of Sensors

**Figure 3:**
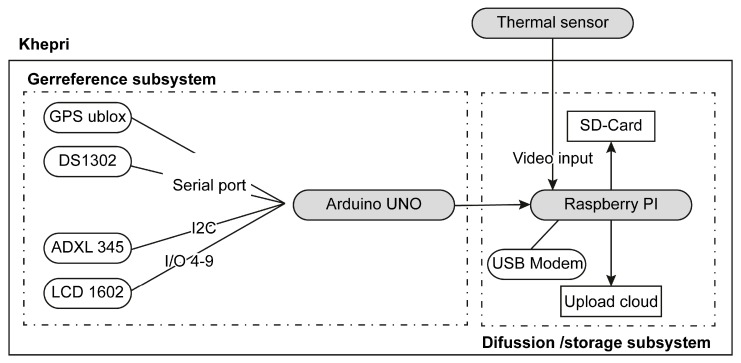
Conceptual model.

Table 1 lists the electronic components and Figure 3 shows a diagram of all the electronic components used in this project. In this study, the Raspberry Pi 2 Model B (Raspberry Pi Foundation, Cambridge, United Kingdom) [44] was used as a single-board computer and the Arduino UNO R3 (Arduino AG, Ivrea, Italy) [45] was used as the microcontroller board. The Raspberry Pi 2 Model B contains a system-on-a-chip (SoC) Broadcom BCM2836 (Broadcom, Irvine, CA, USA), which includes a 900 MHz ARM Cortex-A7, 1 Gb synchronous dynamic random access memory (SDRAM), four Universal Serial Bus (USB) ports, one high-definition multimedia interface port (HDMI), one micro secure digital (SD) card to store information, and one Ethernet port. The Raspberry Pi stores the video registered by the connected sensor. The Arduino UNO R3 is a microcontroller board based on the ATmega328 (Atmel, San José, CA, USA) microcontroller, which manages and arranges the onboard sensor data. It has the minimal requirements for the study, containing 14 digital input/output pins, six of which can be used as pulse width modulation outputs, six analogue inputs, an ATmega328 microcontroller with an 18 MHz crystal oscillator, a USB connection, a power jack, an In-Circuit Serial Programming (ICSP) header, 32 KB of flash memory, 2 KB of SRAM and 1 KB of EEPROM (electrically erasable programmable read-only memory).

A GPS Shield based on the ublox NEO-6M (Ublox, Thalwil, Switzerland) receiver module [46] is used to obtain the coordinates of the device. The positioning engine uses 50 channels and measures time based on Time To First Fix (TTFF) of less than 1 second, searching in parallel both time and frequency space, allowing instant satellite discovery. The horizontal position accuracy is 2.5 m, which is sufficient for this study. The 3-axis digital accelerometer ADXL 345 (SparkFun electronics, Boulder, CO, USA) [47] was used to measure static acceleration as tilt and dynamic acceleration to determine the attitude of the system. It has an output resolution equal to 10 bits and a sensitivity deviation from ideal equal to ±1%. Each data package was temporally referenced with a DS1302 (Maxim Integrated, San José, CA, United States) real-time clock [48]. It operates with a supply of 2 to 5.5 V and can operate between 0 and +70 °C. It contains a real-time clock/calendar and 31 bytes of static RAM. 

A USB modem was used to upload information generated by the system to the cloud to be diffused to other users. Finally, a LCD Screen 1602 (Shenzhen Eone Electronics CO., Ltd., Shenzhen, China) [49] was used to display battery percentage and the verification of all the components. It operates with a supply of 5 V. A rechargeable 7.4 V LiPo Battery with 1400 mAh capacity was used to power the system.

All the electronic components were distributed inside a polylactic acid (PLA) case printed by a 3D printer. PLA material is a biodegradable polymer harder than other materials such as acrylonitrile–butadiene–styrene (ABS), melting at lower temperatures (from 180 to 220 °C), with a glass transition temperature around 60 °C. The PLA case dimensions are 132 × 132 × 47 mm and its weight is equal to 50 gr. The total weight of the device is 375 gr in addition to the thermal sensor weight, in this case, 263 gr. 

### 2.2. Technical Implementation

As Figure 3 shows, the Khepri is composed of two main subsystems: the Arduino and the Raspberry Pi connected to an external sensor. The Arduino subsystem manages geo-information data packages generated by GPS and Inertial Measurement Unit (IMU). The GPS Shield is connected to digital port 0 and 1 of the Arduino UNO board. From the NMEA (National Marine Electronics Association) string, received latitude and longitude are extracted. This information is used to locate the system on a cartograph in real time. An accelerometer ADXL 345 is connected to digital port 2 and analogical port 4 and 5 of the Arduino UNO board. Data from ADXL 345 are converted to yaw, pitch and roll angles, allowing the orientation of the system to be known. Both the thermal sensor and the Khepri work simultaneously, displaying their respective data packages on screen. Using the coordinates obtained by GPS and orientation by IMUs, the Khepri’s location is approximately (≈ 1m) registered to indicate the approximate location of the thermal sensor. As such, a user will be able to see, with an external device, live onscreen thermal video in a window along with its geolocation on a cartograph in another window. 

As the data is being rendered, the Raspberry Pi collects the information from the thermal sensor and from the Arduino subsystem and makes a backup copy on a SD card. From the thermal sensor, a thermographic video is stored while the Arduino system creates a log file with geolocation information, which allows the video to be georeferenced for later use if needed. 

Two methods have been adopted for the cartographic display: API Google Maps and Leaflet. Public API Google Maps is a set of object classes, functions and data structures applied using JavaScript or other scripting languages [50]. The latest version is supported by traditional web browsers or those implemented on mobile devices. API Google Maps uses cartography from Google Maps. On occasion, the resolution of this cartography is insufficient, either in temporal or spatial terms. For this reason, a web mapping application was developed using the JavaScript library Leaflet [51]. In this research project, an orthomosaic was produced using RGB images registered by an UAV flight over a CSP plant with a spatial resolution equal to 5 cm. This orthomosaic was published through a Web Map Service (WMS) using Geoserver [52] and displayed by Leaflet. With the availability of these options, users will be able to choose the most suitable mapping source.

In short, the Khepri will display three windows: one with thermographic information, the second with a map viewer and the third with a virtual horizon. These windows will be distributed on the screen. 

To diffuse information from sensors or devices onboard UAVs in real time, most applications use a radio frequency transmission system that connects the UAV with a Ground Control Station (GCS) [53,54]. Images or video registered by the onboard sensor are transmitted to the GCS and further relayed to other users. On occasion, a lost transmission data link can occur and result in the loss of information sent from the UAV to the GCS [55]. In this developed solution, the system itself has the function of sending the information in real time without needing a GCS to communicate with the users as the Khepri transmits the information of interest over the Internet via USB modem. Therefore, it is necessary to have a solid mobile data link to work in real time, which is maintained in these plants for security reasons. In other cases, the inspection can be analyzed later using the information registered in the SD card.

**Figure 4:**
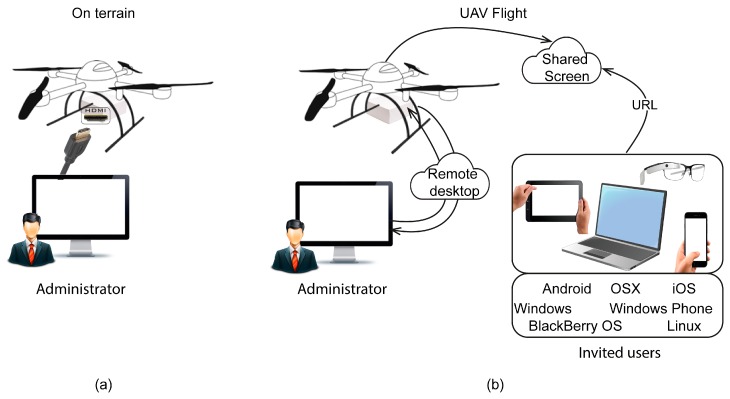
User profiles and cases of use: (**a**) The administrator sets the system on terrain and (**b**) The system onboard an unmanned aerial vehicle (UAV) flight being accessed by the administrator through a remote desktop and invited users with different operating systems accessing inspection via the URL.

Two user profiles have been defined for the system: administrator and invited user. The administrative user has system configuration privileges, for example, to control how the thermal sensor works or to interrupt data transmission. These profiles can be applied in two different scenarios. In the first (Figure 4a), while the system is on terrain, the administrator can connect a screen to the Khepri through the HDMI port, accessing the windows and functions of the system. In the second scenario, the administrator can connect remotely, accessing the Khepri through Chrome Remote Desktop using a Chrome browser with an Internet connection (Figure 4b). In this case, if there are invited users, they will be able to access the thermal inspection. This is because the system, when the administrator chooses, shares the desktop screen live using the application Screenleap (Figure 4b). Invited users are linked via Uniform Resource Locator (URL) for view-only access, which allows the system to be accessible regardless of the type of device and/or operating system. This solution guarantees the interoperability of devices and operating systems. It is also worth noting that the number of invited users is unlimited. This multi-access to the Khepri allows users to work at different locations and with different objectives simultaneously. For example, the UAV operator can perform the flight with focus on guaranteeing the security of the UAV mission while, simultaneously, other users are managing the thermal inspection, whether at the same plant or in another country. Moreover, this activity can be performed by more than one user to guarantee the quality of the visual analysis. 

## 3. Results

**Figure 5:**
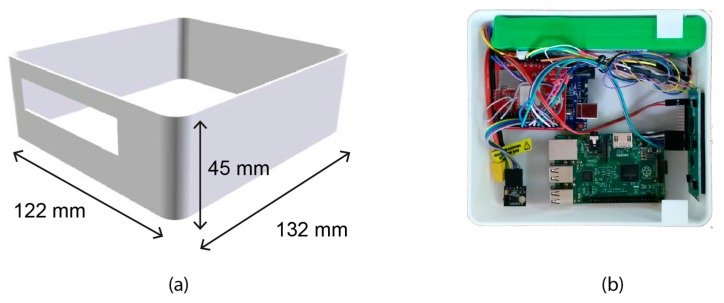
Polylactic acid (PLA) case: (**a**) Design of prototype and (**b**) Distribution of components.

The prototype PLA case measures 132 × 122 × 45 mm, which allows it to be installed in an UAV gimbal (Figure 5a). It was designed to be ergonomic, resistant to deformation and low weight. All the components of the Khepri were distributed in such a way that the center of mass was close to the center of the PLA case (Figure 5b).

**Figure 6:**
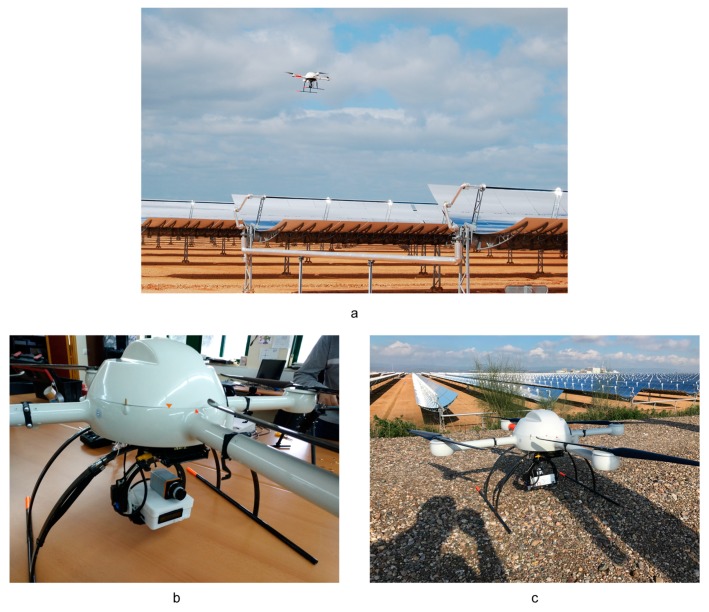
(**a**) The UAV performing an inspection on a concentrated solar power (CSP) plant; (**b**) Front view of the system onboard the UAV; (**c**) Back view of the system onboard.

An UAV flight campaign over a CSP plant was executed with two objectives: to verify that the system works properly, and to test different altitudes Above Ground Level (AGL) and cruising speeds to define the specifications for use. A quadrocopter, model MD4-1000 (Microdrones GmbH, Siegen, Germany), was used as the UAV to perform the flights above the La Africana PTC CSP plant, located in Córdoba, Spain (CSP plant details in [56]) (Figure 6a). This CSP plant has 104 km of absorber tubes distributed on the surface. Currently, at this CSP plant, a thermographic inspection is performed by a technician with a thermal gun walking through the solar field and inspecting the absorber tubes. Once a problem is detected, the incident and its location are noted. With this methodology, an inspection takes one week for an expert technician and two if the inspection is done by a junior technician, and is only performed once a month or every two months. These numbers highlight the ineffectiveness of the method. 

MD4-1000 is a vertical take-off and landing platform that is entirely carbon designed and equipped with 4 × 250 W gearless brushless motors powered by a 22.2 V battery. Maximum cruising and climb speeds are 12 and 7.5 m/s, respectively. MD4-1000 can operate in remote control mode or automatically. The payload was the thermographic sensor Gobi 640 GiGe (Xenics nv, Leuvem, Belgium) connected to the Khepri (Figure 6b,c). The Gobi 640 GiGe is an uncooled micro-bolometer sensor that operates in the 8–14 μm spectral band with an image size of 640 × 480 pixels and a focal length of 18 mm. It is calibrated to operate from −25 to 125 °C with a resolution equal to 50 mK. Figure 6b,c show a front and back view of the Gobi 640 GiGe and the Khepri onboard the UAV.

**Figure 7:**
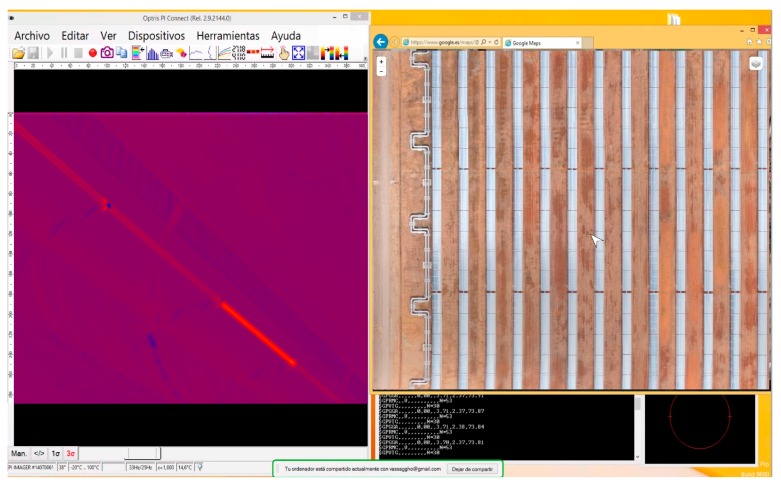
Screen details from Khepri during a thermal inspection on a CSP.

Figure 7 shows a screen capture from Khepri during a thermal inspection on the PTC CSP plant. The left window contains the information from the thermal sensor, which shows an anomaly on an absorber tube. Simultaneously, in the right window, the system is located on an orthomosaic of the CSP plant. In this example, an orthomosaic produced by an RGB sensor onboard an UAV and published by Leaflet is used to position the system.

**Figure 8:**
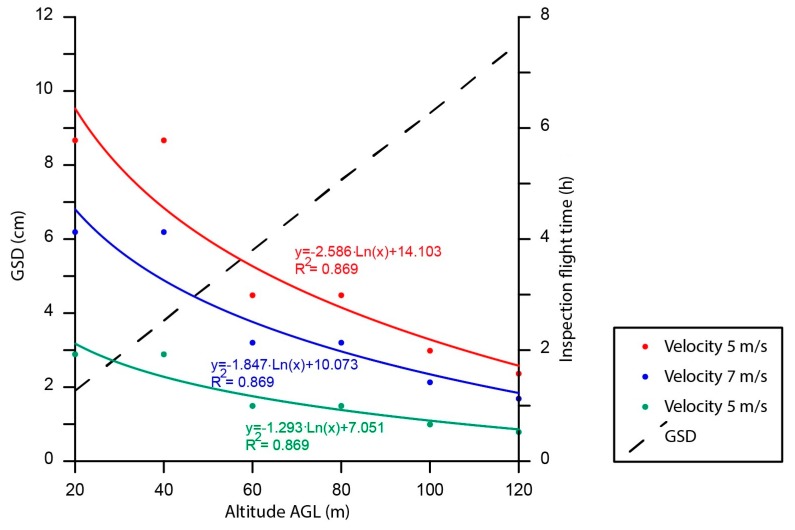
Inspection flight time considering cruising speed and altitude AGL.

In this study, a set of flight missions were flown at altitudes of 20, 40, 60, 80, 100 and 120 m AGL. The UAV flew in autonomous mode with each flight mission uploaded previously. Each altitude AGL is linked to a specific ground sample distance (GSD) value (Table 2). Additionally, three different cruising speed settings were used: 5, 7 and 10 m/s. Maximum velocity was set to ensure the video did not undergo blurring caused by platform movements. All flights were performed under the same conditions, the wind speed being equal to 1 m/s. Table 2 summarizes, for each altitude AGL, the GSD of the video frame and, for each cruising speed, the time, in hours, taken to inspect the entire plant. Duration is expressed apart from the time spent taking off and landing and time to change batteries. In this study, the GSD ranged from 1.9 cm × pixel^−1^ at 20 m AGL, to 11.3 cm × pixel^−1^ at 120 m AGL. Inspection duration ranged from 0.8 h flying at 120 m AGL and cruising speed equal to 10 m/s, to 5.8 h flying at 20 m AGL and cruising speed equal to 5 m/s. Table 2 demonstrates that, as altitude AGL increases, inspection duration decreases because each video frame covers more area, monitoring several lines of absorber tubes simultaneously in each lap. This occurs regardless of the cruising speed of the UAV. Altitude AGL and duration inspection show a logarithmic correlation for all cruising speeds equal to 0.869 (Figure 8). Therefore, as altitude AGL was increased, inspection duration logarithmically decreased. In addition, cruising speed was not relevant in detecting anomalies during the inspection due to the great temperature differential of a broken absorber tube.

**Figure 9:**
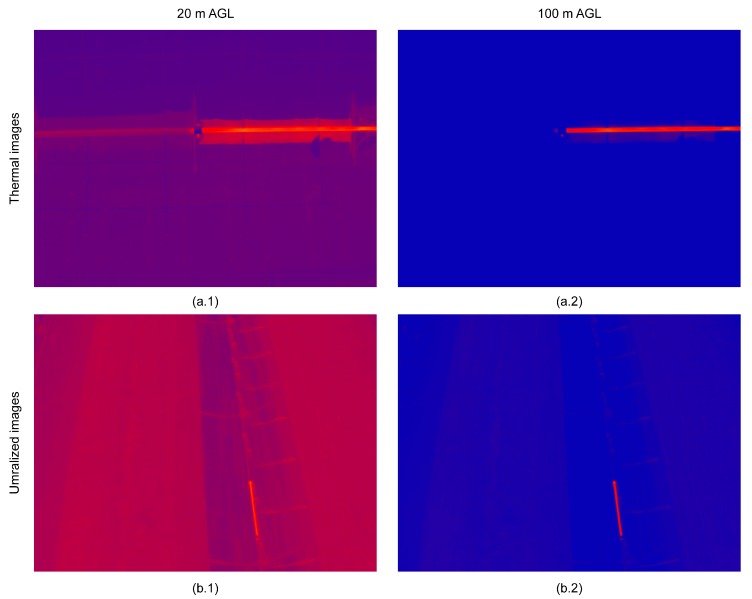
Examples of the UAV inspection at (**a**) 20 m and (**b**) 100 m AGL; (**1**) un-stretched and (**2**) stretched histograms.

As for visual inspection, Figure 9 shows an example of a video frame at 20 m AGL (Figure 9(a1,a2)) and 100 m AGL (Figure 9(b1,b2)). All considered altitudes AGL detected heat losses from absorber tubes satisfactorily. However, due to the wide range of inspection times, it is necessary to optimize the flight parameters for best results. All positive inspections were caused by the high temperature around the broken absorber tubes. Flying at 20 m AGL (Figure 9(a1)), the thermographic video showed bellows between absorber tubes, making it possible to identify the thermal section along the tube. This high level of detail requires longer UAV flight times at low altitude AGL. Increasing altitude AGL (Figure 9(b1)) diffuses the image, but the anomaly is still detected, and inspection time is decreased. In both cases, if the histograms of the frames are stretched, the anomalies in absorber tubes are clearly shown independently of altitude AGL (Figure 9(a2,b2)).

In summary, as a strategy in a PTC CSP plant inspection, the system can fly at low and high altitude AGL jointly, optimizing UAV flights and therefore time inspection. Firstly, a UAV flight can be executed at a high altitude AGL, detecting absorber tubes which show anomalies, as well as obtaining the percentage of anomalous tubes of the entire CSP plant. Once these are located, they can be viewed at very high spatial resolution and analyzed with precision by flying at low-altitude AGL in specific areas. Using this strategy, it is possible to optimize the number and duration of UAV flights without losing information.

All tests were observed in real-time by different users in different locations and with different devices. The invited users were located both at the CSP plant and our university department. The invited users followed the inspection simultaneously using tablets and smartphones with android and iOS operating systems, laptops with Windows, Linux and OSX and android smartglasses. To connect to the inspection using mobile devices, a QR code was provided with the URL where the Khepri windows were being displayed. Connection tests were successful, with no interruptions occurring during the connection. The administrative profile interacted with the Khepri both directly, before the UAV flights started, and from our university department through a connection by remote desktop. 

Considering how van Blyenburgh [18] established that UAVs ought to be used for activities which are “dull, dirty and dangerous”, and walking inspections are categorically dull, recurring mode UAV flights at a PTC CSP plant fits van Blyenburgh’s defined use while at the same time intensifying the cadence of thermal inspection at the plant. Table 3 shows the percentage of improvement in inspection time with the developed system due to its ultra-high resolution and speed compared to current methods based on walking inspections at the CSP plant. The improvement of time inspection ranges from 85.6% flying at 20 m AGL and a cruising speed of 5 m/s to 98.0% flying at 120 m AGL and cruising speed equal to 10 m/s. Independently of altitude AGL and cruising speed used, the developed system allows a more productive method without losing information compared to current methods. 

## 4. Conclusions

A system onboard an UAV to monitor CSP plants using OSH and OSS was developed with customization capabilities depending on use. The thermal inspection of a CSP plant was successful at different altitudes AGL and cruising speeds, and allowed the possibility to observe the inspection using different devices with different operating systems. As a result, we propose flights at two altitudes AGL. Firstly, UAV flights at 100–120 m AGL can locate incidents on absorber tubes around the plant. Once they are located, a focused inspection at low-altitude AGL, 20 m AGL, can be performed to analyze the problem in detail. With this strategy, inspection time is optimized. 

The proposed methodology is more efficient than traditional methods based on walking with a thermal gun through the solar plant. Moreover, the proposed system will allow the numbers of inspections to be increased, improving the efficiency of CSP plants. 

The entire system has been developed using OSH and OSS, allowing for customization depending on application. In the future, RGB or multispectral sensors will be able to be used in other scenarios such as precision agriculture. Moreover, board computers with higher capacities will be able to be used on the Khepri to process video or images in real time to detect anomalies and send alerts, among other possible uses. This automation would permit a reduction in the number of experts required to analyze the thermal video to detect leaks.

## Figures and Tables

**Table 1:** Khepri electronic components.

Title 1	Weight (gr)	Dimension (mm)
Raspberry Pi 2 Model B	45	85.6 × 56.5
Arduino UNO R3	25	68.6 × 53.4
GPS Shield ublox NEO-6M	32	61.4 × 53.3 × 16
Accelerometer ADXL 345	5	3 × 5 × 1
DS1302 clock	10	9.91 × 7.87 × 4.45
Modem USB	24	88 × 28 × 10
LCD Screen 1602	50	80 × 36 × 13.5
Lipo Battery	79	125 × 7 × 21

**Table 2:** Summary of the unmanned aerial vehicle (UAV) flights.

		UAV Inspection Time (Hours)		
Altitude AGL ^1^ (m)	GSD ^2^ (cm)	5 m/s	7 m/s	10 m/s
20	1.9	5.8	4.1	2.9
40	3.8	5.8	4.1	2.9
60	5.7	3.0	2.1	1.5
80	7.6	3.0	2.1	1.5
100	9.4	2.0	1.4	1.0
120	11.3	1.6	1.1	0.8

^1^ Altitude AGL: Altitude above ground level. ^2^ GSD: Ground sample distance.

**Table 3:** Improved productivity of UAV inspection work versus manual inspection.

Altitude AGL (m)	5 m/s (%)	7 m/s (%)	10 m/s (%)
20	85.6	89.7	92.8
40	85.6	89.7	92.8
60	92.5	94.7	96.3
80	92.5	94.7	96.3
100	95.0	96.4	97.5
120	96.1	97.2	98.0
